# Poorly differentiated synovial sarcoma is associated with high expression of enhancer of zeste homologue 2 (EZH2)

**DOI:** 10.1186/1479-5876-10-216

**Published:** 2012-10-30

**Authors:** Yi-Che Changchien, Péter Tátrai, Gergő Papp, Johanna Sápi, László Fónyad, Miklós Szendrői, Zsuzsanna Pápai, Zoltán Sápi

**Affiliations:** 1 1st Department of Pathology and Experimental Cancer Research, Semmelweis University, Üllői út 26, Budapest, H-1085, Hungary; 2 Institute of Enzymology, Research Center for Natural Sciences, Hungarian Academy of Sciences, Budapest, Hungary; 3 Department of Control Engineering and Information Technology, Budapest University of Technology and Economics, Budapest, Hungary; 4 Department of Orthopedics, Semmelweis University, Budapest, Hungary; 5 Military Hospital-State Health Centre Department of Oncology, Budapest, Hungary

**Keywords:** Synovial sarcoma, EZH2, Polycomb group, Histone methyltransferase, H3K27me3

## Abstract

**Background:**

Enhancer of zeste homologue 2 (EZH2) is a polycomb group (PcG) family protein. Acting as a histone methyltransferase it plays crucial roles in maintaining epigenetic stem cell signature, while its deregulation leads to tumor development. EZH2 overexpression is commonly associated with poor prognosis in a variety of tumor types including carcinomas, lymphomas and soft tissue sarcomas. However, although the synovial sarcoma fusion proteins SYT-SSX1/2/4 are known to interact with PcG members, the diagnostic and prognostic significance of EZH2 expression in synovial sarcoma has not yet been investigated. Also, literature data are equivocal on the correlation between EZH2 expression and the abundance of trimethylated histone 3 lysine 27 (H3K27me3) motifs in tumors.

**Methods:**

Immunohistochemical stains of EZH2, H3K27me3, and Ki-67 were performed on tissue microarrays containing cores from 6 poorly differentiated, 39 monophasic and 10 biphasic synovial sarcomas, and evaluated by pre-established scoring criteria. Results of the three immunostainings were compared, and differences were sought between the histological subtypes as well as patient groups defined by gender, age, tumor location, the presence of distant metastasis, and the type of fusion gene. The relationship between EZH2 expression and survival was plotted on a Kaplan-Meier curve.

**Results:**

High expression of EZH2 mRNA and protein was specifically detected in the poorly differentiated subtype. EZH2 scores were found to correlate with those of Ki-67 and H3K27me3. Cases with high EZH2 score were characterized by larger tumor size (≥ 5cm), distant metastasis, and poor prognosis. Even in the monophasic and biphasic subtypes, higher expression of EZH2 was associated with higher proliferation rate, larger tumor size, and the risk of developing distant metastasis. In these histological groups, EZH2 was superior to Ki-67 in predicting metastatic disease.

**Conclusions:**

High expression of EZH2 helps to distinguish poorly differentiated synovial sarcoma from the monophasic and biphasic subtypes, and it is associated with unfavorable clinical outcome. Importantly, high EZH2 expression is predictive of developing distant metastasis even in the better-differentiated subtypes. EZH2 overexpression in synovial sarcoma is correlated with high H3K27 trimethylation. Thus, along with other epigenetic regulators, EZH2 may be a future therapeutic target.

## Background

Synovial sarcoma, an aggressive soft tissue tumor with high rate of local recurrence and distant metastasis, is currently thought to originate from mesenchymal stem cells [1]; hence, the traditional term ‘synovial’ is a misnomer. Synovial sarcomas occur most commonly in young patients, representing about 10% of soft tissue sarcomas in all age groups and about 15-20% in adolescents, with more than 80% of the cases arising in deep soft tissues around large joints or tendons. Synovial sarcomas can display monophasic (MPSS), biphasic (BPSS) and poorly differentiated (PDSS) histology, with the latter accounting for approx. 10% of the cases. PDSS is defined by high cellularity, high nuclear grade, and high mitotic activity, as well as areas of necrosis. Its morphology is typically dominated by small round cells or rhabdoid-like cells similar to undifferentiated embryonic cells, and its clinical course tends to be aggressive with early recurrence and metastasis [2,3].

Enhancer of zeste homologue 2 (EZH2) is a member of the polycomb group (PcG) protein family. The PcG family consists of epigenetic transcriptional repressors which participate in cell cycle regulation, DNA damage repair, cell differentiation, senescence, and apoptosis. PcG regulation is known to be involved in the maintenance of stem cell signature, but also in tumor development [4]. Specifically, EZH2 acts as a histone methyltransferase targeting the N-terminal tail of histone 3 and producing a characteristic trimethylated H3-Lys27 (H3K27me3) motif. It shows high expression in cells possessing embryonic gene expression signature, while its amount declines with tissue maturation and differentiation [5]. Abnormal overexpression of EZH2 has been reported in a wide variety of tumor types including carcinomas, lymphomas, cutaneous melanoma, and soft tissue sarcomas [6]. High expression of EZH2 is generally associated with advanced stages of tumor progression, aggressive tumor behavior, and dismal clinical outcome [7].

Intriguing hypotheses have recently been formulated on the collaboration between EZH2 and SYT-SSX, the chimeric gene diagnostic of synovial sarcoma. The chromosomal translocation t(X;18)(p11;q11) can be demonstrated in over 95% of cases by fluorescence *in situ* hybridization (FISH) or real-time PCR (RT-PCR) and produces one of the fusion genes SYT-SSX1, SYT-SSX2 or, rarely, SYT-SSX4 [1,8]. Due to its intranuclear localization but lack of a chromatin binding domain, SYT-SSX is thought to modify gene expression by associating with sequence-specific DNA-binding proteins [9]. SYT has been described to interact with transcription-enhancing trithorax group proteins such as the SWI/SNF chromatin remodeling complexes via its SNH domain, while SSX has been shown to bind with the transcription-silencing PcG proteins such as EZH2 via its SSXRD domain. SYT-SSX is hypothesized to bring together these oppositely acting protein complexes, allowing each to make its contribution to sarcomatogenesis [10,11]. Identification of possible target genes influenced by this epigenetic deregulation has begun, but much effort is still needed to elucidate the pathomechanism in full detail [12].

Although high EZH2 expression was shown to be generally associated with poor prognosis in soft tissue sarcomas [13], neither differential expression of EZH2 in the various histological subtypes of synovial sarcoma nor the association of EZH2 with H3K27 trimethylation, tumor behavior, and clinical parameters has been investigated in this particular tumor type. Therefore, a tissue microarray-based immunohistochemical study was designed to address these points. Since synovial sarcoma patients are divided into low-risk and high-risk prognostic groups based on age (younger or older than 25 years), tumor size (larger or smaller than 5 cm), mitotic activity, and the presence or absence of poorly differentiated areas [3,14], correlations were sought between EZH2 expression and these prognostic factors, as well as with other clinical data such as gender, tumor location, distant metastasis, and the type of fusion gene which also has been reported to impact disease outcome [15]. The impact of EZH2 expression on overall survival was analyzed on a Kaplan-Meier curve. EZH2 expression was also measured at the mRNA level by quantitative real-time PCR (qRT-PCR) to support the immunohistochemical findings.

## Methods

### Tissue specimens and microarrays

We constructed TMAs containing duplicates of 6-mm cores from 55 cases of previously diagnosed synovial sarcoma. Our samples included 6 PDSS, 39 MPSS, and 10 BPSS tissues fixed in 10% formalin and embedded in paraffin. Tumor tissues were selected from the archives of the 1^st^ Department of Pathology and Experimental Cancer Research, Semmelweis University, Budapest, Hungary, from the years between 1996 and 2009, and sampled by anexpert soft tissue pathologist (Z.S.). The patients gave informed consent to the research purpose use of their tissue. Only primary tumors without preoperative chemo- or radiotherapy were chosen. Clinical data (age, gender, tumor location, tumor size, presence or absence of metastases and the type of the fusion gene) were obtained from the institutional records. Clinical follow-up data were also available for 32 cases (time after operation: 8–162 months). Pathological diagnoses were made according to the World Health Organization (WHO) classification [2], and confirmed by either FISH or RT-PCR. The research was conducted in concordance with the Institutional Ethical Guidelines.

### Immunohistochemistry

After preparing 4-μm cuts from the formalin-fixed, paraffin-embedded TMAs, sections were deparaffinized in xylene and rehydrated in a descending ethanol series. Antigen retrieval was achieved by using either Bond Epitope Retrieval Solution 1 (pH~6) or Bond Epitope Retrieval Solution 2 (pH~9) (Leica Microsystems, Wetzlar, Germany) at 99–100°C for 20–30 minutes. Monoclonal mouse anti-EZH2 (1:25, clone 11, BD Biosciences, USA), monoclonal rabbit anti-trimethyl-Histone H3 Lys27 (1:200, clone C36B11, Cell Signaling Technology, USA), or monoclonal mouse anti-Ki-67 (1:50, clone MIB-1; Dako, Denmark) antibodies were applied on the slides. Immunohistochemical staining was performed on a Leica BOND-MAX™ autostainer (Leica Microsystems, Berlin, Germany), and peroxidase/DAB Bond™ Polymer Refine Detection System (Leica Microsystems) was used for visualization.

### Scoring system

**Figure 1:**
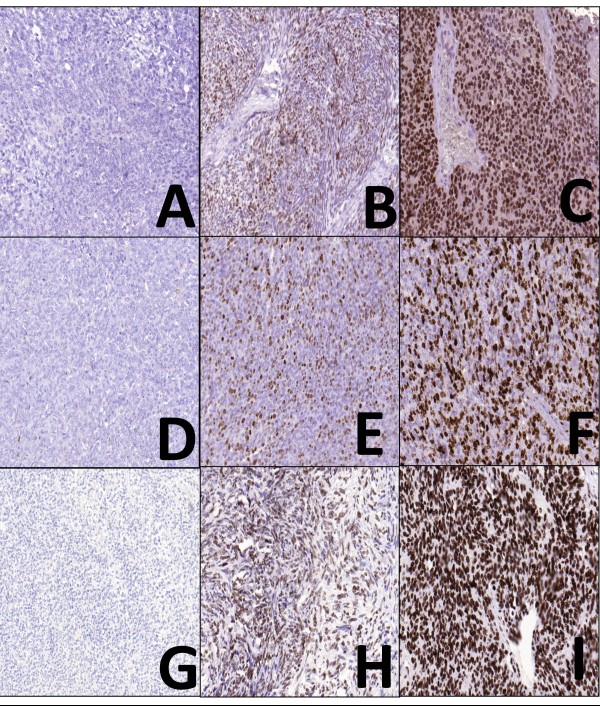
Photomicrographs representing the scores 0, 3, and 6 for EZH2, H3K27me3, and Ki-67 immunostaning.

To assess the immunohistochemical labeling of EZH2, H3K27me3, and Ki-67, immunostained slides were evaluated under a 10x magnification objective. Nuclear staining intensity was scored as follows: 0, no visible staining; 1, weak; 2, moderate; 3, strong. Higher score was chosen if at least 30% of positive tumor cells showed stronger intensity. To quantify the extent of immunostaining, the percentage of tumor cells with positive nuclear reaction was counted, and a score was assigned as follows: 0, no visible staining; 1, 1–50%; 2, 51–75%; 3, over 75%. Each core contained at least 100 tumor cells to be evaluated. The two scores were summed to yield a final score ranging from 0 to 6. Fields of view representative of scores 0, 3, and 6 are shown in Figure 1. A total score ≤ 3 was defined as low and ≥ 4 as high.

### Analysis of EZH2 mRNA expression

For EZH2 gene expression analysis, total RNA was isolated from formalin-fixed, paraffin-embedded blocks of synovial sarcoma tissue by using RecoverAll™ Total Nucleic Acid Isolation Kit (Ambion, Austin, USA). The quality of isolated RNA was adequate for gene expression analysis in 13 MPSS, 2 BPSS and 6 BPSS cases. cDNA was generated from 1 μg of total RNA using High Capacity cDNA Reverse Transcription Kit (Applied Biosystems, Foster City, USA), following the instructions of the supplier. Quantitative real-time PCR (qRT-PCR) was performed in a LightCycler 480 Real-Time PCR System (Roche Applied Science, Indianapolis, USA) by using ABI TaqMan Gene Expression Assay for human EZH2 gene (assay ID: Hs01016789_m1; Applied Biosystems) according to the manufacturer’s protocol. The expression of EZH2 was normalized to endogenous human ribosomal protein S18 (assay ID: Hs02387368_g1; Applied Biosystems), and cDNA from lymph node served as calibrator. Results were obtained as crossing point (Cp) values. Expression levels were calculated by using the 2^-ΔΔCp^ method.

### Statistical analysis

Prism 4 software (GraphPad, USA), SigmaPlot and SigmaStat software packages (SPSS, version 11.0, IBM, USA) and the VassarStats website ( http://www.vassarstats.net) were used for statistical analyses. Kruskal-Wallis test was used for the comparison of more than two groups, while pairwise comparison of non-Gaussian data sets was done by the Mann–Whitney test. Correlations were analyzed by the Spearman’s rank order correlation test (Spearman's rho, ρ) and coefficient of determination (R^2^). Kaplan-Meier curves were created based on the duration of survival after operation, and groups were compared with univariate analysis using the log-rank test. For all analyses, P values <0.05 were considered as statistically significant.

## Results

### Clinical data

The clinical characteristics of our 55 synovial sarcoma cases and the results of immunostaining are summarized in Additional file 1: Table S1. Six tumors were classified histologically as poorly differentiated, while 39 were described as monophasic and 10 as biphasic. The numbers of male and female patients were 31 and 24, respectively. Age younger than 25 years was recorded in 8 cases, while 47 patients were older than 25 years. The mean age was 47 (range, 18–79). The tumor was located on the periphery in 39 cases and centrally in 16 cases. Tumors were larger than 5 cm in 14 cases. Distant metastasis was present in 31 cases. There were 35 cases associated with SYT-SSX1 fusion gene and 20 cases with SYT-SSX2.

### High expression of EZH2 and high abundance of H3K27me3 in PDSS

**Figure 2:**
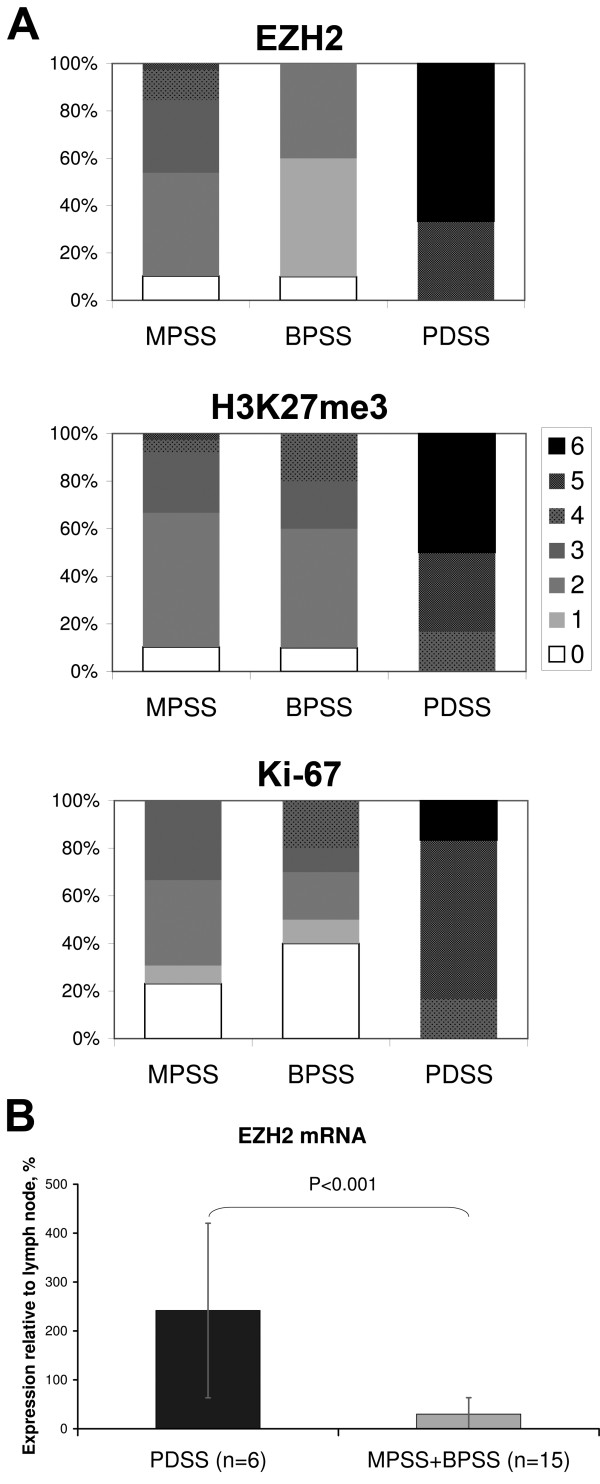
**Distribution of scores 0–6 in the MPSS, BPSS, and PDSS subtypes, shown as stacked columns (A).** mRNA expression of EZH2 in the PDSS vs. MPSS+BPSS groups, mean Â± S.D. Human ribosomal protein S18 was used as endogenous control and human lymph node was served as calibrator.

Percent distribution of immunohistochemical scores is illustrated in Figure 2A, and statistical results are summarized in Table 1. Similar to Ki-67, high immunohistochemical scores of EZH2 and H3K27me3 were specifically recorded in PDSS and only rarely in the other subtypes. Overexpression of EZH2 in PDSS relative to MPSS and BPSS (8.1x higher expression in PDSS, P<0.001) was also confirmed at the mRNA level (Figure 2B). Significant differences between PDSS, MPSS and BPSS for EZH2, H3K27me3 and Ki-67 immunohistochemical scores were detected by Kruskal-Wallis test (all P<0.001). The mean scores of all three markers were significantly higher in PDSS as compared with MPSS and BPSS. Furthermore, scores of EZH2 and H3K27me3, but not of Ki-67, were significantly higher in patients with larger tumor size, and all three markers were significantly higher in those with distant metastasis (Table 1). No statistically significant differences in mean immunohistochemical scores were found with regard to clinical factors such as age, gender, tumor location, or the type of fusion gene. Thus, EZH2 and H3K27me3 may be regarded as auxiliary markers of the poorly differentiated subtype, although the potential of EZH2 and H3K27me3 immunostaining to discriminate between PDSS and the other subtypes was inferior to that of Ki-67 (sensitivities, specificities, and positive predictive values for EZH2: 1.00, 0.82, 0.4; H3K27me3: 1.00, 0.90, 0.54; Ki-67, 1.00, 0.96, 0.75, respectively).

**Table 1:** Comparison of immunohistochemical scores across patient groups sorted by histological subtype or clinical / molecular characteristics

***Compared groups***	***Statistical test used***		***Result***
**EZH2**			
PDSS and MPSS and BPSS	Kruskal-Wallis		P < 0.001
PDSS vs. MPSS	Mann–Whitney		P < 0.001
PDSS vs. BPSS	Mann–Whitney		P = 0.001
MPSS vs. BPSS	Mann–Whitney		NS
**H3K27me3**			
PDSS and MPSS and BPSS	Kruskal-Wallis		P < 0.001
PDSS vs. MPSS	Mann–Whitney		P < 0,001
PDSS vs. BPSS			P = 0.001
MPSS vs. BPSS	Mann–Whitney		NS
**Ki-67**			
PDSS and MPSS and BPSS	1		P < 0.001
PDSS vs. MPSS	Mann–Whitney		P < 0,001
PDSS vs. BPSS	Mann–Whitney		NS
*Compared groups*	*EZH2*	*Ki-67*	*H3K27me3*
**Gender**			
Male vs. female	NS	NS	NS
**Age**			
≤25 y/o vs. >25 y/o	NS	NS	NS
**Location**			
Central vs. peripheral	NS	NS	NS
**Tumor size**			
<5cm vs. ≥5cm	P < 0.001	NS	P = 0.032
**Distant metastasis**			
Present vs. absent	P < 0.001	P = 0.021	P = 0.003
**Fusion gene**			
SYT-SSX1 vs. SYT-SSX2	NS	NS	NS

(PDSS: poorly differentiated synovial sarcoma; MPSS: monophasic synovial sarcoma; BPSS: biphasic synovial sarcoma; NS: not significant).

**Figure 3:**
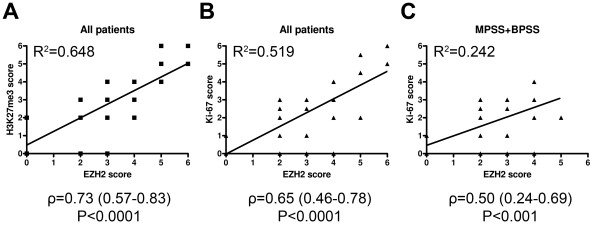
**Scatter graphs with linear trend lines indicate positive correlations between EZH2 and the other markers.** All patients were included in **(A)** and **(B)**, while PDSS patients were excluded in **(C)**. Spearman’s *r* (ρ) and coefficient of determination (R^2^) values are shown at the bottom.

Across all subtypes, EZH2 expression was found to correlate with the abundance of H3K27me3 motifs (Figure 3A, ρ: 0.73, P<0.0001, R^2^=0.648), indicating causal relationship between EZH2 activity and the presence of the associated epigenetic marker in synovial sarcoma. Significant positive correlation could also be demonstrated between EZH2 and Ki-67 scores both when including all cases (Figure 3B, ρ: 0.65, P<0.0001, R^2^=0.519) and with the exclusion of PDSS cases (Figure 3C, ρ: 0.50, P<0.001, R^2^=0.242), suggesting that EZH2 expression and proliferative activity were positively linked in the better-differentiated subtypes as well.

### EZH2 as a potential prognostic marker in synovial sarcoma

**Figure 4:**
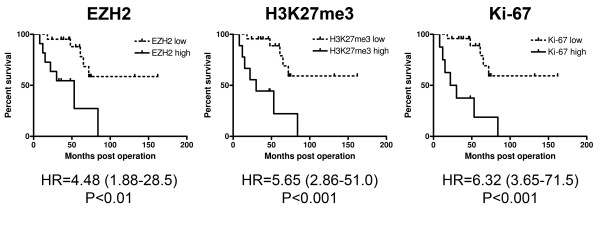
**Kaplan-Meier survival curves for high vs. low EZH2, HeK27me3, and Ki-67 score.** Hazard ratios (HR) of the high-score groups with 95% confidence intervals are shown at the bottom.

Kaplan-Meier curves generated by separating patients on the basis of high versus low EZH2 and H3K27me3 scores were similar to the one based on Ki-67 score (Figure 4). However, Ki-67 was a superior predictor of tumor-associated death, since the hazard ratios referring to high EZH2, H3K27me3, and Ki-67 expression were 4.48, 5.65, and 6.32, respectively. Nevertheless, high EZH2 score also proved to be a valuable predictor of disease outcome, since it was significantly associated with larger tumor size and the presence of distant metastasis. Moreover, these associations held true not only in the entire patient population but also after the exclusion of PDSS cases. In contrast, high H3K23me3 failed to show such associations, and high Ki-67 was associated with larger tumor size in all patients only (Table 2) indicating that EZH2 may be useful in the stratification of MPSS and BPSS patients into low and high risk prognostic groups with respect to the likelihood of developing distant metastasis.

**Table 2:** Association of high EZH2, H3K27me3, and Ki-67 scores with the risk of large tumor size and distant metastasis (NS: non-significant)

	**Relative Risk**	**95% Confidence Interval**	**P value**
**High EZH2 score**			
*All Cases*			
-Tumor size >5 cm	4.09	1.78-9.40	P<0.001
-Distant metastasis	2.20	1.49-3.23	P<0.001
*Cases other than PDSS*			
-Tumor size >5 cm	4.00	1.63-9.82	P=0.02
-Distant metastasis	2.09	1.36-3.21	P=0.02
**High H3K27me3 score**			
*All Cases*			
-Tumor size >5 cm	2.25	1.07-4.73	NS
-Distant metastasis	1.64	1.09-2.46	NS
*Cases other than PDSS*			
-Tumor size >5 cm	1.12	0.20-6.17	NS
-Distant metastasis	1.20	0.55-2.60	NS
**High Ki-67 score**			
*All Cases*			
-Tumor size >5 cm	2.62	1.29-5.34	P=0.04
-Distant metastasis	1.71	1.17-2.51	NS
*Cases other than PDSS*			
-Tumor size >5 cm	1.75	0.39-7.88	NS
-Distant metastasis	0.98	0.24-4.03	NS

## Discussion

In our study, high expression of EZH2 was predominantly found in the poorly differentiated histological subtype of synovial sarcoma, which was associated with aggressive clinical behavior. High levels of EZH2 were shown to be associated with poor clinical outcome in other tumor types as well, and the mechanisms that link EZH2 activity with tumor progression are gradually being unfolded [7]. Yet the exact causes and consequences of EZH2 overexpression in PDSS remain to be clarified. With regard to its transcriptional regulation, a hypothetic role can be assigned to MYC, since recent gene expression profiling data revealed up-regulation of genes located on chromosome 8q, including MYC, in PDSS [16], and MYC has been reported to induce EZH2 in prostatic carcinoma [17]. EZH2 expression may also be triggered by hypoxia, a condition present in nearly all solid tumors: HIF1α-dependent transactivation of EZH2 was demonstrated in breast cancer-initiating cells [18]. Furthermore, direct induction of EZH2 by the EWS-FLI1 fusion protein in Ewing’s sarcoma suggests that translocation-associated chimeric proteins may also play a regulatory role [19]. At the post-transcriptional level, microRNAs are likely to modulate EZH2 levels, since EZH2 is a validated target of the promyogenic miR-26a, and high expression of EZH2 was consistently paralleled by suppression of miR-26a in rhabdomyosarcoma [20].

Once overexpressed, EZH2 places epigenetic marks that prevent RNA polymerase II-dependent transcriptional elongation and lead to silencing of the downstream genes. PcG family members are arranged into multimeric polycomb repressive complexes (PRC), and EZH2 is a core member and catalytic unit of PRC2. H3K27me3 produced by PRC2 is recognized by PRC1 which, in turn, monoubiquitylates lysine 119 of histone H2A. PRC2 also interacts with other repressive epigenetic modifiers such as histone deacetylases (HDAC) and DNA methyltransferases (DNMT) which promote chromatin condensation [7,21,22]. Remarkably, the binding of PRC1 hinders the access of other chromatin remodeling complexes such as SWI/SNF that may have transcription-enhancing functions [23], which implies that out of the antagonistic partners of SYT-SSX in synovial sarcoma, CpG may ultimately dominate over SWI/SNF [10].

Although the target genes of EZH2-mediated silencing in synovial sarcoma still wait to be identified, EZH2 activity is generally thought to favor the conservation of undifferentiated state and give way to rapid proliferation. Significantly overlapping sets of genes were found to be targeted by PRC2 in prostatic carcinoma and embryonic cells, and repression of these genes was associated with poor clinical outcome [24]. EZH2 is therefore believed to drive tumor cells into a more aggressive, embryonic stem-like state, as it is clearly exemplified by EZH2-overexpressing tumors with embryonic morphology like rhabdomyosarcoma or Ewing’s sarcoma [25]. EZH2 also facilitates cell cycle progression: its expression is induced by E2F, a chief coordinator of mitotic entry, while EZH2 itself represses, among others, the tumor suppressor INK4/ARF and the pro-apoptotic regulator Bim [6,26,27]. Our findings indicate that the link between EZH2 expression, high mitotic activity, and undifferentiated morphology exists in synovial sarcoma as well, since EZH2 scores strongly correlated with those of Ki-67 and were highest in poorly differentiated tumors.

Another positive correlation found in our study, namely the one between EZH2 expression and the abundance of H3K27me3 motifs, could be logically expected from the catalytic activity EZH2 is known to exert in PRC2. The activating somatic mutation Y641 of EZH2 leads to high H3K27 trimethylation in lymphomas [28,29], and high levels of H3K27me3 consequent to EZH2 hyperactivity have been reported in hepatocellular carcinoma and esophageal squamous cell carcinoma [30,31]. It is all the more intriguing why in certain tumors, such as carcinomas of the breast, ovary, and pancreas, no clear correlation between EZH2 expression and H3K27 trimethylation was found; rather, quite counterintuitively, both high EZH2 and low H3K27me3 turned out to have adverse prognostic significance [32]. Explanations proposed for this apparent discrepancy include the disruption of PRC2 by overproduced EZH2, the formation of tumor-specific PRCs with different histone substrate specificity, and Akt-mediated inhibitory phosphorylation of EZH2 [32-34].

By examining associations between EZH2 expression, histological subtype, and clinical factors such as tumor characteristics and disease course, we wished to clarify whether EZH2 (and/or H3K27me3) immunohistochemistry may provide any additional diagnostic, prognostic, or therapeutic information that cannot be deduced from other data. The markers investigated herein showed significant association with histology and distant metastasis, but varied independently from other clinical factors and the type of fusion gene. EZH2 and H3K27me3 scores also exhibited significant association with tumor size. Although Ki-67 distinguished more accurately between PDSS and the better-differentiated subtypes, both high EZH2 and high H3K27me3 were preferentially associated with PDSS. Further, whereas Ki-67 as a well-established prognostic marker in soft tissue sarcomas proved to be a superior predictor of overall survival [35], high EZH2 status – but not high H3K27me3 or high Ki-67 – was found to be predictive of distant metastasis in the MPSS+BPSS group. Thus, while not sufficiently specific when applied alone, both EZH2 and H3K27me3 can be used as auxiliary immunohistochemical markers of the poorly differentiated subtype in doubtful cases (e.g., better-differentiated histomorphology coupled with high mitotic rate, or vice versa). Moreover, EZH2 status, along with other recently proposed factors such as ploidy [36], may refine the current stratification of MPSS and BPSS patients into low- and high-risk subgroups, thus influencing prognosis and possibly also the therapeutic decisions.

Lastly, EZH2 as a highly expressed pro-oncogenic regulator may also be an attractive candidate target for the future therapy of synovial sarcoma. SYT-SSX closely collaborates with both EZH2 and HDAC in the repression of the tumor suppressor early growth response 1 (EGR1) [10]. Romidepsin (FK228), a HDAC inhibitor, reactivated EGR1 expression and caused tumor shrinkage in a preclinical synovial sarcoma model, presumably by disrupting the interactions within this complex [12,37]. It is reasonable to assume that concomitant inhibition of HDAC and EZH2 might yield a synergistic effect. Successful repression of EZH2 was achieved by small interfering RNA (siRNA) as well as using the small-molecule pharmacologic inhibitor 3-deazaneplanocin A in neuroblastoma cells . Translation of these results into the treatment of synovial sarcoma may open novel therapeutic [38]opportunities.

## Conclusion

High expression of EZH2 helps to distinguish poorly differentiated synovial sarcoma from the monophasic and biphasic subtypes, and it is associated with unfavorable clinical outcome. Importantly, high EZH2 expression is predictive of developing distant metastasis even in the better-differentiated MPSS and BPSS subtypes. EZH2 overexpression in synovial sarcoma is correlated with high H3K27 trimethylation, indicating a functional participation of EZH2 in PRC2. In summary, EZH2 can be used as an auxiliary diagnostic and prognostic marker in the histopathologic evaluation of synovial sarcoma in addition to the markers currently in use. Later, overexpressed EZH2 may become a therapeutic target in synovial sarcoma, especially when inhibited in combination with other pro-oncogenic epigenetic modulators.

## Abbreviations

BPSS: Biphasic synovial sarcoma; DNMT: DNA Methyltransferase; EZH2: Enhancer of zeste homologue 2; FISH: Fluorescence in situ hybridization; H3K27me3: Trimethyl-histone H3 (Lys27); HDAC: Histone deacetylase; MPSS: Monophasic synovial sarcoma; PcG: Polycomb group; PDSS: Poorly differentiated synovial sarcoma; PRC: Polycomb repressor complex; qRT-PCR: Quantitative real time- polymerase chain reaction; siRNA: Small interfering RNA.

## Competing interests

The authors declare that they have no competing interests.

## Authors’ contributions

*YC* evaluated the immunohistochemical reactions, collected and analyzed the results, and designed and drafted the article. *PT* critically revised the manuscript and helped with the preparation of figures. *GP* performed the RT-PCR and helped in drafting the manuscript. *JS* performed and validated the statistical data. *LF* collected clinical information and helped with the preparation of figures. *MS* contributed the surgical specimens. *ZP* contributed the clinical follow up data. *ZS* conceived and coordinated the work, and helped in drafting the manuscript. All authors have read and approved the final manuscript.

## Supplementary Material

Additional file 1 **Table S1.** Clinical data, immunohistochemical scores, and EZH2 gene expression values of 55 synovial sarcoma cases. Legend: gender: M: male; F: female; size: 0, <5cm; 1, ≥5cm; location: P, peripheral; C: central; end point: 1, deceased; 0, alive at the indicated time point (months post op.); NA: data not available. Click here for file
